# Intracranial Germinomas: Diagnosis, Pathogenesis, Clinical Presentation, and Management

**DOI:** 10.1007/s11912-023-01416-2

**Published:** 2023-04-10

**Authors:** Natalia Kremenevski, Michael Buchfelder, Nirjhar Hore

**Affiliations:** Department of Neurosurgery, University of Erlangen-Nürnberg, Erlangen, Germany

**Keywords:** Germinoma, Biomarkers, Prognosis, Outcome, Radiotherapy, Chemotherapy

## Abstract

**Purpose of Review:**

Intracranial germinomas constitute a rare brain tumor entity of unknown etiology, characterized by unique histopathology and molecular biology. In this manuscript, we review the literature focusing on the epidemiology, histopathology with molecular biology, clinical presentation with emphasis on tumor location, diagnostic workup, and current treatment strategies with related clinical outcomes of intracranial germinomas.

**Recent Findings:**

Although the optimal treatment strategy remains a matter of debate, intracranial germinomas respond well to radiotherapy, chemotherapy, or a combination of both and are characterized by very high cure and survival rates. It is well-known that early discrimination of germinomas from other intracranial neoplasms facilitates the timely initiation of appropriate treatment, thereby contributing to the reduction of morbidity as well as mortality.

**Summary:**

Ongoing research will need to be directed towards discovering and refining reliable parameters for early diagnosis and evaluation of prognosis in patients with intracranial germinomas.

## Introduction

Central nervous system germ cell tumors are rare brain neoplasms, the etiology of which remains unknown and controversial. They are divided into two heterogenous groups: germinomas and non-germinomatous germ cell tumors [1]. Each tumor entity is characterized by its unique histopathology and molecular biology, individual growth pattern, and response to treatment [2]. Germinomas are the more commonly occurring of the two groups and account for about two-thirds of all intracranial germ cell tumors (iGCT) [3, 4••]. Although the optimal treatment strategy remains a matter of debate, they generally respond well to radiotherapy, chemotherapy, or a combination of both and are characterized by a good survival rate [5–7]. Some patients may benefit from additional neurosurgical resection [8, 9]. Germinomas often occur in the pineal or the sellar-suprasellar regions and less frequently in the basal ganglia/thalamus [3]. Diagnosis is usually based on clinical presentation, imaging findings, and determination of tumor markers. Since they may initially present with non-specific signs of endocrine, hypothalamic, visual, and cognitive disturbances—despite clinical findings otherwise congruent to the location and size of the lesions—precise and especially timely diagnosis can nevertheless be difficult [10•, 11]. Correspondingly, many patients exhibit clinical signs long before a diagnosis is made. To compound matters, even radiological examinations like computed tomography (CT) and magnetic resonance imaging (MRI) often cannot provide evidence of tumor manifestation for years [12]. It is well-known that early discrimination of germinomas from other intracranial neoplasms facilitates the timely initiation of appropriate treatment, thereby contributing to the reduction of morbidity as well as mortality. To this effect, a histological confirmation of germinomas by stereotactic or endoscopic biopsy may be necessary [13]. Additionally, late side effects of the radiotherapy have attracted increasing attention in recent decades due to the consequently high survival rate [14, 15]. In this manuscript, we would like to present a review of the literature focusing on the epidemiology, histopathology with molecular biology, clinical presentation with emphasis on tumor location, diagnostic workup, and current treatment strategies with related clinical outcomes of intracranial germinomas.

## Epidemiology

Even in the current era of population-based cancer registries, the incidence and the distribution patterns of intracranial germinoma remain ill-defined. One of the reasons certainly lies in their rarity. Other reasons include differences in demographic characteristics as well as in genetic und environmental risk factors between countries. Furthermore, the collection of information about rare cancers sometimes requires the inclusion of additional data sources, a process often significantly hampered by their paucity. With an incidence of 60–77%, germinomas account for about two-thirds of iGCTs [16–18]. Characterized by a gender-specific predisposition in terms of site of growth, the area of most common manifestation of germinomas in males is the pineal region (75%) and in females the sellar/suprasellar region (75%) [3, 19]. There has been a considerable variationreported in iGCTs frequencies between races and countries. iGCTs account for 0.4–3.3% of all children and adolescent primary brain tumors in North America and Europe, whereas this incidence is five to eight times higher in Japan, China, and South Korea [16–20].

According to recent published data of the CBTRUS Statistical Report on Primary Brain and Other Central Nervous System Tumors Diagnosed in the USA in 2014–2018, the overall incidence rate (IR) of iGCTs was 0.08 per 100,000 person-years (py) with a clear predominance of the male gender (IR for males = 0.12 and for females = 0.04 per 100,000 py, respectively) [21•]. Moreover, the IR for iGCTs for Asian and Pacific Islanders (0.13 per 100,000 py) exceeded those observed for other races. The previous CBTRUS data from 2008–2012 to 2011–2015 has reported a higher overall IR of iGCTs (0.10 per 100,000 py) as well as a higher IR in males [20, 21•]. The IR of germinomas was 0.058 per 100,000 py, and the average and median age at diagnosis was 18 and 16 years, respectively.

McCarthy et al. [16] published contradictory results comparing the incidence of iGCT between Japan and the USA. Of the four databases analyzed, two registries represented 26% of the population of the USA and the other two represented 31.8% of the total population of Japan. There was no statistically significantly difference in the IR of iGCT between the two countries (IR of 0.096 per 100,000 py for Japan and 0.075 per 100,000 py for the USA). The authors also found an equal gender-based tumor distribution.

However, the Lee SH et al.’s [22] study has shown the IR of iGCTs (0.179 per 100,000 py) in South Korea to be more than twice as high as in the USA. The population-based epidemiologic studies from Kumamato and Miyazaki Prefectures in Japan have found the same age-adjusted IR of iGCTs (0.18 and 0.17 per 100,000 py, respectively) [23, 24]. These results support the assumption that East Asian populations have a higher incidence of iGCTs than others.

## Histopathology

Germinomas represent the malignant correlate of a normal embryonic stage of development. The tumor appears to be similar in their macro- and microscopic, as well as immunohistochemical characteristics to testicular seminomas and ovarian dysgerminomas [25]. Due to the frequently deep locations of germinomas in the brain and tiny biopsy specimens, it is difficult in some cases to achieve an accurate histopathological diagnosis.

**Fig. 1:**
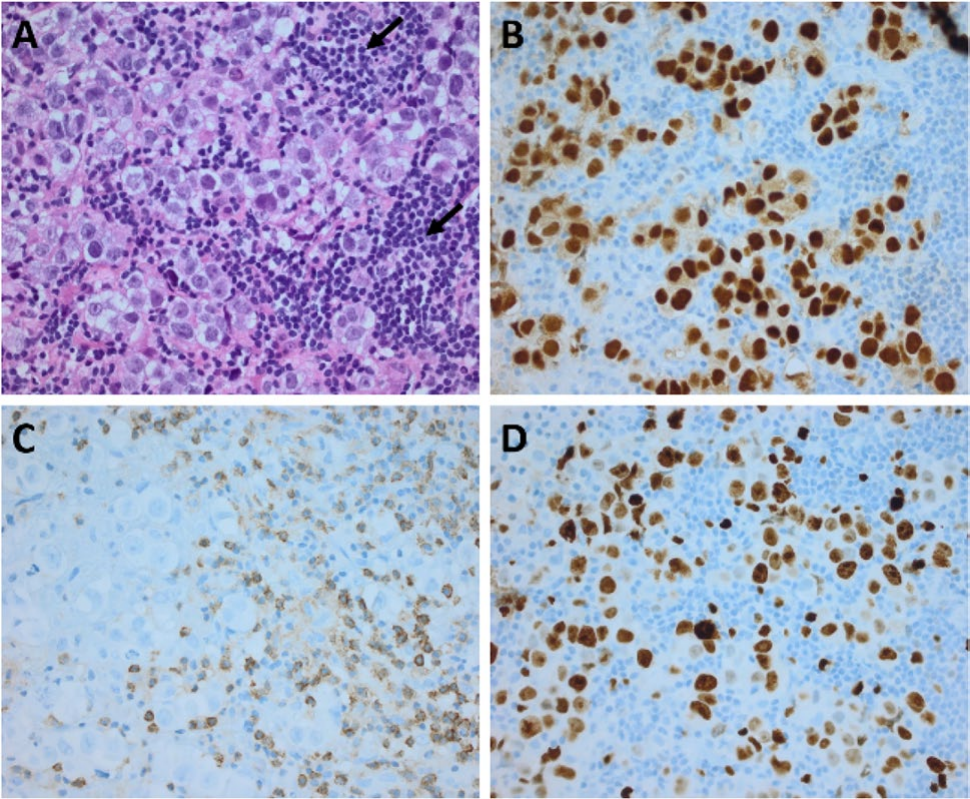
**A** HE staining shows a tumor with large, round nuclei and a clear cell cytoplasm. Intermingled lymphoid cells (arrows). **B** The germinoma cells with a nuclear expression of OCT4. **C** Interspersed lymphocytic cells (CD45 immunohistochemistry). **D** The tumor shows a high proliferation activity (Ki-67 immunohistochemistry)

Cross-sectional pathology shows a tumor with well-circumscribed solid and soft tissues. The cut surface of the tumor may show a grey-pink color and occasionally have small cysts. There are no areas of necrosis or hemorrhage. Histological examination of germinomas reveals large polygonal undifferentiated cells enclosed in fibrous connective tissue. The cells have high mitotic activity, and their growth occurs in sheets, lobules, or nests rather than single-type patterns. Neoplastic cells are usually characterized by a pale eosinophilic or clear cytoplasm with centrally placed, large and vesicular nuclei and enlarged irregular nucleoli [26–28]. Furthermore, germinomas are well-known to be highly immunogenic tumors initiating autoimmune reactions with subsequent recruitment of immune cells. The infiltrating lymphocytes lying along neoplastic cells create a typical histological appearance described in the literature as a “two-cell pattern” [29] (Fig. 1).

The immunohistochemical staining for specific markers expressed by germinomas provides additional information. It allows confirmation or exclusion of the presence of other germ cell tumors within the tumor tissue samples [30]. There are several specific and sensitive immunohistochemical markers consistently expressed and recorded in germinomas. D2-40 (podoplatin), c-kit (transmembrane protein with tyrosine kinase activity), and OCT 3/4 (octamer-binding transcription factor 3/4) have often been used to establish the diagnosis of a germinoma [31–33]. Other discriminative markers such as SALL4 (sal-like protein 4), LIN28A (RNA-binding protein LIN28), and CD30 (tumor necrosis factor receptor) can be evaluated to facilitate the differential diagnosis [34, 35]. Diffuse cytoplasmic and cell membrane labeling for placenta alkaline phosphatase (PLAP), normally expressed by primordial germ cells, is also helpful in identifying tumor cells [36]. The representative histological and immunohistochemical images from intracranial germinomas are shown in Fig. 1.

## Pathogenesis and Molecular Biology

The pathogenesis and molecular biology of germinomas have remained a topic of substantial debate. Historically, Friedman and Moore introduced the term germinomas after examination of testicular tumors [37]. Many years later, Teilum G. formulated the so-called “germ cell theory” stating that all iGCTs originate from the malignant transformation of primordial germ cells during embryonic development [38, 39]. Although other explanations of the etiology of germ cell tumors have been postulated, Teilum’s theory has enjoyed widespread acceptance and has been incorporated into the brain tumor classification of the World Health Organization [1]. Comprehensive global research efforts have led to the discovery of specific pathways and signaling networks of germinomas and to an improved understanding of tumor pathogenesis. Epigenetic reprogramming via a transcriptional regulation mechanism through DNA hypomethylation of some genes, for example, cancer germline antigen genes, occurs during germ cells development [40, 41]. Fukushima et al. found an extensive genome-wide low DNA methylation in germinomas in comparison with other subtypes of intracranial germ cell tumors [42].

Chromosomal studies have revealed gains in 1p,2q,4q, 8p,11p, 12p, and 21q and losses in 1p/q, 3p/q, 4p, 5q, 9p/q, 10p/q 11q/p, 13q, 15q, 16p, 17q, 18p/q 19p/q, and 20p [4••, 40, 43]. A frequent XXY genotype abnormality was reported, and germinomas were also noted to exhibit exclusively intracranial manifestation in patients with Klinefelter syndrome [44]. Takami et al. from the Japanese iGCT Consortium found the gain in 2q and 8q and loss of 5q, 9p/q, 13q, and 15q to be correlated with worse outcomes [4••].

Furthermore, somatic mutations in KIT and RAS genes were identified and found to be “mutually exclusive” in most germinomas [40]. These mutations lead to alterations of the mitogen-activated protein kinase (MAPK) or phosphoinositide 3-kinase (PI3K) pathways, both of which play an important role in the regulation of cell cycle, metabolism, apoptosis, differentiation, and migration. Although Fukushima et al. considered these pathways to be an important mechanism in the development of germinomas, their effects on prognosis and outcome remain unknown [40]. Moreover, Ichimura et al. compared patient survival data with detected alterations along the MAPK or PI3K pathways and were not able to show a statistically significant difference in survival [45]. There are speculations considering wide experience with other cancers whether specific targeting inhibition of these pathway/s might be a part of the further germinomas research topic.

## Clinical Presentation

Displaying an infiltrative growth pattern, germinomas are characterized by a wide spectrum of clinical manifestations. Symptoms include general weakness, irritability, headache, nausea, vomiting, mental status changes, seizures, endocrine abnormalities, disturbance of sexual development, and isolated cases of stunted growth [46, 47]. Slow tumor progression may hamper diagnosis, with successful correlation between often subjective symptoms taking from weeks to years [48]. Germinomas often occur in adolescents and young adults, and the vagueness of symptoms is frequently assumed to be psychosomatic in nature, leading in turn to delayed diagnosis and treatment [3]. Tumor location, size, and extend of adjacent tissue involvement correlate most closely with clinical presentation [49].

### Sellar Germinomas

Clinical signs and symptoms of sellar germinomas are secondary to the development of many endocrine abnormalities associated with the combination of infiltrative or destructive tumor growth and mass effect in the sellar region and neighboring structures [46, 50]. It has been reported in the literature that germinomas cause a greater incidence of endocrinopathy than craniopharyngiomas due to destruction or dysfunction of the hypothalamus and pituitary gland [51]. Prolactin secretion is increased, although galactorrhea may not be present in case of severe hypothalamic dysfunction due to an absence of sex hormones necessary for milk production. Furthermore, other endocrine deficiencies leading to stunted growth, precocious puberty, or delayed sexual development, as well as menstrual irregularity, have been observed. Diabetes insipidus (DI) has been found in more than 90% of germinomas cases in the sellar region, which is why patients often seek medical attention due to a protracted history of polyuria and polydipsia [52]. Other common presenting complaints include headache, nausea, or vomiting. Although the appearance of visual disturbances including narrowing of visual field and decreased visual acuity is another important symptom caused by compression or invasion of the optic chiasm, some patients may not be aware of any visual impairment until later during the diagnostic workup [53]. The reported incidence at diagnosis varies from 40 to over 90%. Moreover, fatigue and loss of appetite, altered sleep patterns, behavioral problems, and poor scholastic performance can manifest as simultaneously presenting symptoms leading to significant diagnostic delay.

### Pineal Germinomas

The clinical manifestations of germinomas in the pineal region are determined by the anatomical relationship between the pineal groove and adjacent intracranial structures [3, 19]. Patients usually have a shorter duration of complaints since even a small neoplastic pineal lesion can lead to compression of the cerebral aqueduct. Initial presenting signs and symptoms are characteristic of elevated intracranial pressure secondary to obstructive hydrocephalus like headache, nausea, vomiting, and visual disturbance. The neurological signs and symptoms involve impairments such as vertigo, dyskinesia or ataxia, and general weakness [12, 54]. A characteristic symptom constellation caused by the compression midbrain structures has been reported in the literature as Parinaud’s syndrome and consists of upward gaze paralysis, light-near dissociation, and convergence-retraction nystagmus [55]. Approximately 75% of the patient with tumors located in the pineal region present with Parinaud’s syndrome [54]. Seizures and behavioral changes have been seen in another 25% of patients.

### Basal Ganglia and Thalamus Germinomas

The basal ganglia and thalamus germinomas, as opposed to germinomas growing in sellar and pineal regions, present in an insidious manner and are characterized by slow progression. Due to the discrepancy between tumor size and severity of clinical manifestation, duration from symptom onset to definitive diagnosis varies from 1 month to several years [12, 56]. The first signs of tumor manifestation in this region appear to be cognitive abnormalities and changes in mental status. The involvement of the extrapyramidal system can be responsible for slowly progressive hemiparesis, dystonia, rigidity, bradykinesia, or dyskinesia [54]. Endocrine abnormalities are usually not observed in these tumor locations.

## Diagnostic Workup

### Magnetic Resonance Imaging Characteristics of Germinomas

MRI represents the imaging modality of choice in the diagnosis of suspected central nervous diseases, providing high-resolution delineation of pathological lesions, their location, and anatomical relationships with neighboring structures [10•, 11, 57, 58]. Although CT can also be used in the diagnostic imaging workup, MRI appears to be the preferred modality due to higher sensitivity compared to CT, especially at initial presentation [59, 60].

**Fig. 2:**
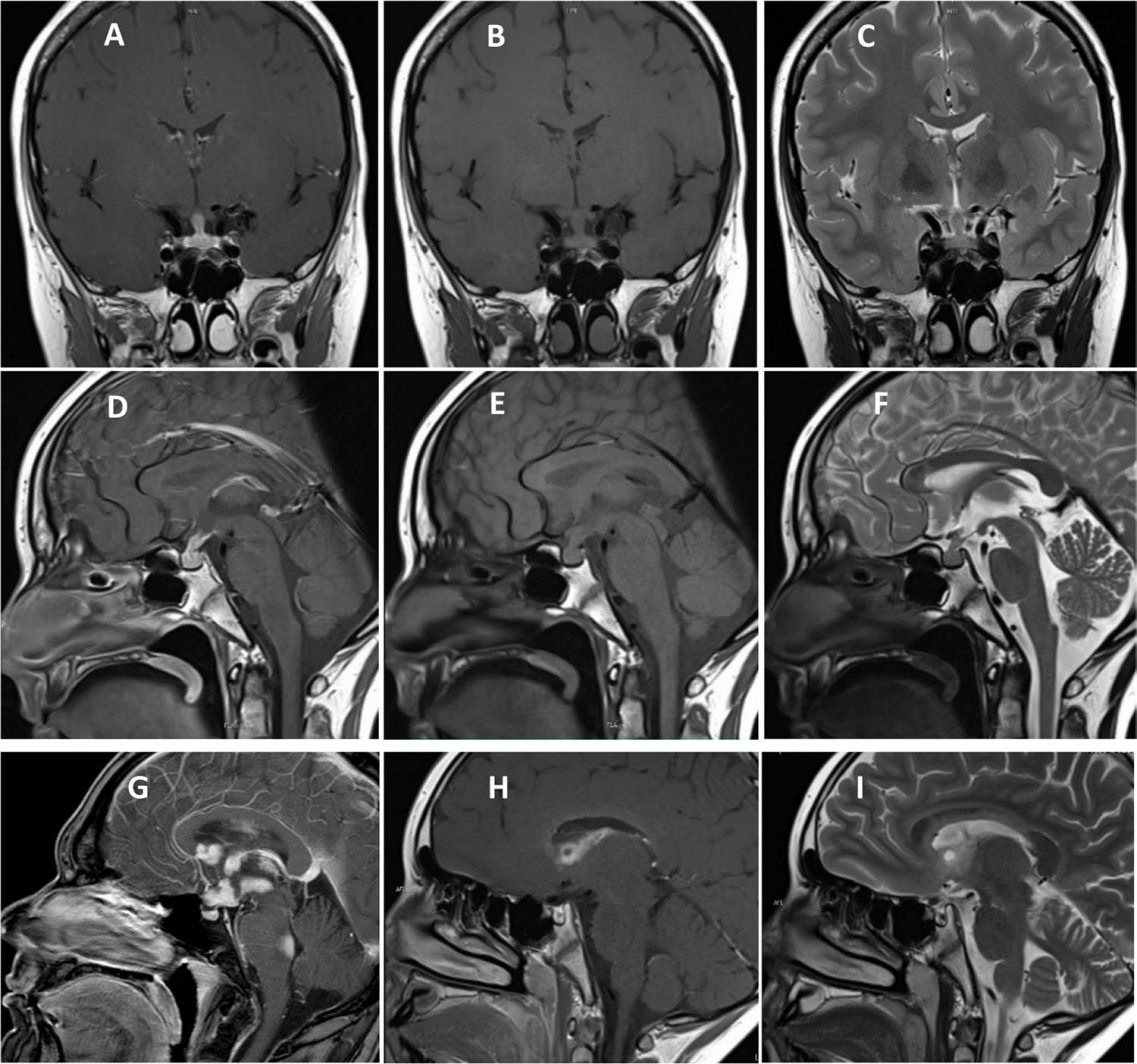
Typical magnetic resonance imaging of intracranial germinoma. Coronal (**A**–**C**) and sagittal (**D**–**F**) contrast-enhanced T1-weighted (**A**, **D**), T1-weighted (**B**, **E**), and T2-weighted (**C**, **F**) images shown a germinoma involving the pituitary stalk. Contrast enhanced T1-weighted sagittal image shows a disseminated germinoma (**G**). Contrast-enhanced T1-weighted and T2-weighted images show a solid germinoma with a cystic component located in the right lateral ventricle wall (**H**, **I**)

Germinomas tend to appear on MRI scans as well-demarcated round, square-round, or oval masses. Large lesions may display cystic and necrotic changes as well invade the adjacent brain parenchyma. The tumors are generally hypointense to isointense on T1-weighted images and isointense to hyperintense on T2-weighted images. There is uniform or inhomogeneous enhancement after intravenous gadolinium administration (Fig. 2). Perifocal edema may be observed in some cases. Calcification and small cysts in the pineal tissues can often be seen [57, 58]. Ipsilateral hemisphere atrophy as a result of thalamo-cortical pathway disruption is highly suggestive of basal ganglia germinomas [59].

Germinomas of the sellar region can initially present on MRI scans as an isolated thickening of the pituitary stalk, thereby posing a significant diagnostic challenge and mandating close neuroradiological follow-up [60]. At an advanced stage, they may appear as a mass with an ill-defined margin and irregular shape and may even displace the optic chiasm.

### Biomarkers of Germinomas

Produced in higher amounts by cancer cells, tumor markers have been known for decades as biochemical tumor indicators. The tumor markers are not used independently for initial tumor diagnostic workups, since their level in plasma or other body fluids can significantly vary between similar neoplastic lesions depending on some factors such as a rate of growth, stage of differentiation, and cellular heterogeneity within the tumor. However, they have remained a valuable diagnostic tool in tumor confirmation, following response to therapy and as an early indicator of tumor recurrence [61].

The evaluation of serum and cerebrospinal fluid placental alkaline phosphatase (PLAP) has shown it to be a reliable biochemical indicator for germinomas [62]. Some studies reported the feasibility of determination of PLAP in the cerebrospinal fluid (CSF) in germinomas in comparison to other intracranial tumors [63]. Other studies confirmed the relationship between high PLAP levels and the presence of germinomas [64]. The sensitivity and specificity of PLAP in distinguishing germinomas from other neoplasms depend on the cutoff value according some publications [65, 66]. Furthermore, the determination of alpha-fetoprotein (AFP) and beta-human chorionic gonadotropin (hCG-ß) levels in serum and CSF may be particularly helpful in the initial differentiation between germinomas and non-germinomatous germ cell tumors [67]. AFP tends to be negative in serum and CSF and has subordinate significance in the diagnostic of germinomas. hCG-ß level should be measured in both serum and CSF and has only appeared in a small portion of germinomas [68]. There is a controversy concerning its prognostic significance. Some earlier studies have been found no difference in the prognosis of patients with normal or high hCG-ß level, whereas more recent studies have demonstrated an association of high hCG-ß level with tumor aggressiveness and poor outcome [69].

All told, in conjunction with other diagnostic procedures, tumor markers are invaluable in the confirmation of diagnosis [70•]. The dynamic changes in tumor marker levels during follow-up may reflect response to the therapy or tumor recurrence [64, 69].

### Endocrine Dysfunction

DI seems to be most frequently reported endocrinological disturbance, often persisting in the post-treatment period [46]. The tumor can invade the floor and walls of the third ventricle as well as the infundibulum and may cause irreversible damage to the neurohypophysis [51]. Besides DI, hormonal dysfunction may involve growth, thyroid-stimulating, luteinizing, follicle-stimulating, and adrenocorticotrophic hormones [52, 53]. On the other hand, hypopituitarism has often occurred in patients with germinomas even though it located in the pineal region [46].

## Treatment

### Radiotherapy

Germinomas belong to a group of highly radiosensitive tumors. The historical gold-standard treatment has been and remains whole-brain or whole ventricular radiotherapy with craniospinal irradiation (CSI) to decrease the risk of spinal metastases with an additional boost to the tumor bed [6]. Many publications have provided evidence of high control rates with an overall survival rate of 90% at 10 years [4••, 5, 14, 15]. However, the irradiation of such a large craniospinal volume mandated taking into consideration late-onset adverse toxic effects [14, 71]. Therefore, other treatment approaches were introduced to evaluate CSI with reduced doses and fields in comparison to previous practice. Data on radiotherapy was collected in the two prospective clinical trials of the German Cooperative Initiative (MAKEI 83/86 and MAKEI 89) [5]. The germinomas patients were treated with 36 Gy to the craniospinal axis with an additional boost of 14 Gy to the tumor site or with 30 and 15 Gy, respectively. The decreased CSI dose appeared to have the same effectiveness in the treatment of germinomas. In another multicenter prospective European study SIOP CNS GCT 96, the outcome in patients with localized germinomas treated with reduced doses of 24 Gy CSI and 16 Gy to the primary tumor was studied. The authors reported a high efficacy of this treatment with 5-year event-free (EFS) und 5-year progression-free survival (PFS) rates of 0.94 ± 0.02 and 0.97 ± 0.02 [72]. Further evidence in justifying the reduced dose of radiotherapy was published 2005 in the Lancet by Rogers et al. [6]. The meta-analysis based on the reviewed published data showed that there was no significant increase of isolated spinal relapses by using the reduced CSI dose in comparison to the whole CSI dose. At the same time, in their retrospective analysis of 180 germinomas cases from 6 Japanese institutions, Shikama et al. found no benefit of whole CSI for PFS [73]. However, there are many other publications concerning the safety of reduced radiotherapy [15, 74, 75].

### Chemotherapy

Chemotherapy has long been a well-established method of the treatment of testicular and ovarian germ cell tumors [76]. The results of these successful treatments encouraged the exploration of the role of chemotherapy as a possible alternative to radiotherapy in patients with germinomas to avoid the long-term deleterious effects of irradiation, especially in the pediatric and young adult populations. Three multicenter international CNS GCT trials have been conducted to study the feasibility of the chemotherapy-only approach in the treatment of intracranial germinomas [77–79]. In the first study, the protocol consisted of carboplatin, etoposide, and bleomycin. Altogether 45 patients with germinoma were included. It was reported a complete response rate of 84% for germinomas and an EFS of only 42% at a median follow-up of 13 months [77]. The second international CNS GCT trial recruited 19 patients with germinomas. The chemotherapy was modified by exchanging carboplatin with cisplatin and adding a high dose of cyclophosphamide. Three patients died from treatment-related toxicity. The 5-year EFS and OS were only 47% and 68%, respectively [78]. In the third study, the involved patients were treated according to stratified risk with one of two risk-tailored chemotherapy regimens. Pure germinomas received less intensive chemotherapy than germinomas with SGC (syncytiotrophoblastic) components. Here, 11 relapses from twenty-five patients were observed at a mean of 30.8 months [79]. All three international CNS GCT studies brought forward a body of evidence that the intensive chemotherapy-alone approach is less effective than radiotherapy alone or in conjunction with chemotherapy for patients with germinomas.

### Combination of Chemotherapy and Radiotherapy

A lot of attempts have been made to decrease the radiation-induced side effects without compromising OS and EFS. The combination of chemotherapy and irradiation led to the subsequent reduction of the radiotherapy volume and dose. This formed the basis for the prospective studies of the French Society of Pediatric Oncology. In 29 patients enrolled with histologically proven germinomas, the prophylactic CSI was replaced with the chemotherapy and 40 Gy applied to the initial tumor bed [80]. The results of this study with respect to OS and EFS were similar to the treatment strategy with extensive irradiation. The results were confirmed in a second study within a larger patient cohort [81]. Finally, the largest prospective study of a radiation dose reduction strategy in germinomas was published by a Children’s Oncology Group study. This multicenter study enrolled 137 patients across the USA, Canada, and Australia [82••]. The authors applied 18 Gy whole ventricular irradiation (WVI) and a boost of 12 Gy to the tumor bed together with four cycles of carboplatin and etoposide chemotherapy. The outcome analysis showed excellent results with less deterioration of cognition and only 8 relapses among 137 patients [82••]. In recapitulation, the study showed a more favorable outcome in comparison with the radiation only MAKEI study [5] and the combined chemotherapy and irradiation of SIOP CNS GCT 96 [72].

### Role of Neurosurgery in the Treatment of Germinomas

In general, there is a consensus in clinical oncology that besides histological diagnosis, maximum tumor debulking is of critical importance in planning and optimizing further treatment as well as evaluating prognosis of any kind of suspected tumors. In the case of germinomas, however, this was of secondary importance for a long time as neurosurgical interventions were largely restricted to an extended biopsy or treatment of obstructive hydrocephalus [13, 83, 84]. This was probably the case due to their high radiosensitivity and heightened morbidity following open neurosurgical procedures in the suprasellar and pineal regions. Advances in neurosurgical techniques with the introduction of intraoperative neuronavigational MRI systems and cutting-edge surgical microscopes have contributed to a considerably decrease in mortality and morbidity, shorter hospital stays, and improved postoperative quality of life of patients with germinomas [8, 9, 13].

Pineal germinomas can be effectively reached using the infratentorial-supracerebellar route, while endoscopic biopsies can be conducted in pineal tumors with concurrent hydrocephalus [85]. The surgical routes for the suprasellar region include transcranial (pterional or supraorbital craniotomy) and transsphenoidal (direct perinasal, sublabial, or paraseptal) approaches [13, 86].

The extent of influence of neurosurgical intervention on the outcome in patients with germinomas remains unknown, as the critical vascular and neuronal anatomy plays a role on the volume of tumor resection in the pineal, neurohypophyseal, or hypothalamic regions [8, 9]. The pooled data from the International CNS Germ Cell Study Group showed a better outcome in germinomas patients with less than 1.5–2 cm residual tumor [81, 87]. Furthermore, all patients with any residual tumor unresponsive to standard treatment regimens or with MRI-documented tumor progression should undergo a second look surgery.

## Prognosis and Outcome

Due to their sensitivity to radio- and chemotherapy, germinomas have a long and successful treatment history with an excellent prognosis. Radiotherapy alone or in a combination with the chemotherapy resulted in very high cure rates [5, 9, 14, 15, 54]. The longest follow-up series of patients with histologically verified germinomas was published be Matsutani et al. The authors reported an overall survival rate of 91.7%, 87.6%, and 80.2% for 10, 15, and 20 years, respectively [54]. Analysis of the Surveillance, Epidemiology, and End Results database found an OS rate of germinomas of 84.1% and 61.9% of 20 and 30 years, respectively [14]. A review of 96 patients with germinomas from the Intracranial Germ Cell Tumor Consortium showed a 5-year OS rate and PFS of 98.6% and 87.3%, respectively [4••]. Another published retrospective multinational Asian study reported a similar 5-year OS rate and PFS at 97.2% and 89.9%, respectively [75]. A Children’s Oncology Group study from Canada and the USA has recently presented the results of their simplified chemotherapy regime followed by a dose-reduced irradiation trial. This new treatment strategy detailed in a prospective and well-done study demonstrated an exceptional 3-year PFS and OS rate of 94.5% and 100%, respectively [82••].

Furthermore, the prognosis of germinomas depends on tumor location and patient age at diagnosis. Bifocal lesions appear to have a poorer outcome than a solitary one. Adult patients with germinomas (age > 18 years) have a statistically significant poorer OS rate and PFS in comparison with pediatric patients with the same tumor characteristics [88, 89].

Nevertheless, a tenfold increase in mortality was observed in germinoma patients 5 years after completion of treatment in comparison with age-adjusted healthy control subjects [14]. The young age of patients with this tumor entity and long-term survival times have raised concerns about the adverse effects of radio- and chemotherapy in this population group. There are many publications reporting radiation-induced hypothalamic-pituitary dysfunction and musculoskeletal and neurocognitive complications [90, 91]. In their long-term analysis of germinoma treatment, Lee et al. found the development of secondary malignancy in 10 patients (5.3%) with a latency of 20 years (range from 4 to 26 years) [15]. Another group of clinicians documented radiation-induced occlusive vasculopathy of large intracranial arteries and as a consequence increased rate of stroke (11.7% in 16 years after treatment) [92]. They also documented the development of arteriovenous malformations as well as a high rate of secondary neoplasms (16.8%, 19 years after therapy). Moreover, Acharya et al. analyzed the data from a large population-based cancer registry and calculated the cumulative incidence of death due to cancer and subsequent malignancy using a competing risk model [14]. At 25 years, this stood at 16% and 6.0%, respectively, for germinomas survivors.

## Conclusion

Recent publications of the cooperative research groups from Europe, the USA, and Japan have shed new light on the complexities of germinomas, a rare brain tumor entity. There are many works from the Intracranial Germ Cell Tumor Genome Analysis Consortium from Japan addressing the molecular, epigenetic, and genetic pathogenesis of germinomas. Of special note are the results of researchers of the Children’s Oncology Group from North America, who showed in a well-conducted phase II clinical trial that pediatric patients with germinomas can achieve a complete response with a combined treatment consisting of dose-reduced radiation therapy and less toxic chemotherapy. In essence, they demonstrated the feasibility of an optimized therapy strategy directed to reducing treatment—associated long-term side effects with improvement of quality of life in pediatric patients while preserving high cure rates. The application of the specific inhibitors based on solid knowledge of molecular pathways, however, remains a subject of future trials. Ongoing research will need to be directed towards discovering and refining reliable parameters for early diagnosis and evaluation of prognosis in patients with germinomas.
